# Nutritional status and screening tools to detect nutritional risk in hospitalized patients with hepatic echinococcosis

**DOI:** 10.1051/parasite/2020071

**Published:** 2020-12-23

**Authors:** Zhan Wang, Jin Xu, Ge Song, MingQuan Pang, Bin Guo, XiaoLei Xu, HaiJiu Wang, Ying Zhou, Li Ren, Hu Zhou, Jie Ma, HaiNing Fan

**Affiliations:** 1 Department of Hepatopancreatobiliary Surgery, The Affiliated Hospital of Qinghai University Xining 810001 PR China; 2 Qinghai University Xining 810001 PR China; 3 Department of Otorhinolaryngology, The Affiliated Hospital of Qinghai University Xining 810001 PR China; 4 Qinghai Province Key Laboratory of Hydatid Disease Research Xining 810001 PR China; 5 Department of Emergency Surgery, The Affiliated Hospital of Henan University of Science and Technology Luoyang PR China

**Keywords:** Cystic echinococcosis, Alveolar echinococcosis, Nutritional screening tools, Nutritional risk, ESPEN

## Abstract

*Background*: Echinococcosis is a chronic consumptive liver disease. Little research has been carried out on the nutritional status of infected patients, though liver diseases are often associated with malnutrition. Our study investigated four different nutrition screening tools, to assess nutritional risks of hospitalized patients with echinococcosis. *Methods*: Nutritional Risk Screening 2002 (NRS 2002), Short Form of Mini Nutritional Assessment (MNA-SF), Malnutrition Universal Screening Tool (MUST), and the Nutrition Risk Index (NRI) were used to assess 164 patients with alveolar echinococcosis (AE) and 232 with cystic echinococcosis (CE). Results were then compared with European Society for Clinical Nutrition and Metabolism (ESPEN) criteria for malnutrition diagnosis. *Results*: According to ESPEN standards for malnutrition diagnosis, 29.2% of CE patients and 31.1% of AE patients were malnourished. The malnutrition risk rates for CE and AE patients were as follows: NRS 2002 – 40.3% and 30.7%; MUST – 51.5% and 50.9%; MNA-SF – 46.8% and 44.1%; and NRI – 51.1% and 67.4%. In patients with CE, MNA-SF and NRS 2002 results correlated well with ESPEN results (*k* = 0.515, 0.496). Area-under-the-curve (AUC) values of MNA-SF and NRS 2002 were 0.803 and 0.776, respectively. For patients with AE, NRS 2002 and MNA-SF results correlated well with ESPEN (*k* = 0.555, 0.493). AUC values of NRS 2002 and MNA-SF were 0.776 and 0.792, respectively. *Conclusion*: This study is the first to analyze hospitalized echinococcosis patients based on these nutritional screening tools. Our results suggest that NRS 2002 and MNA-SF are suitable tools for nutritional screening of inpatients with echinococcosis.

## Abstract (fr)

*Contexte* : L’échinococcose est une maladie hépatique consommatrice chronique. Il existe peu de recherches sur l’état nutritionnel des patients infectés, bien que les maladies du foie soient souvent associées à la malnutrition. Notre étude a examiné quatre différents outils de dépistage nutritionnel, pour évaluer les risques nutritionnels des patients hospitalisés atteints d’échinococcose. *Méthodes* : Les méthodes Nutritional Risk Screening 2002 (NRS 2002), Short Form of Mini Nutritional Assessment (MNA-SF), Malnutrition Universal Screening Tool (MUST) et Nutrition Risk Index (NRI) ont été utilisées pour évaluer 164 patients atteints d’échinococcose alvéolaire (EA) et 232 avec échinococcose kystique (EK). Les résultats ont ensuite été comparés aux critères de la Société européenne pour la nutrition clinique et le métabolisme (ESPEN) pour le diagnostic de la malnutrition. *Résultats* : Selon les normes ESPEN pour le diagnostic de la malnutrition, 29,2 % des patients avec EK et 31,1 % des patients avec EA étaient malnutris. Les taux de risque de malnutrition pour les patients EK et EA étaient, respectivement : NRS 2002 - 40,3 % et 30,7 % ; MUST - 51,5 % et 50,9 % ; MNA-SF - 46,8 % et 44,1 % ; NRI - 51,1 % et 67,4 %. Chez les patients atteints d’EK, les résultats de MNA-SF et NRS 2002 étaient bien corrélés aux résultats ESPEN (*k* = 0,515, 0,496), et les valeurs de l’aire sous la courbe (ASC) du MNA-SF et du NRS 2002 étaient respectivement de 0,803 et 0,776. Pour les patients atteints d’EA, les résultats NRS 2002 et MNA-SF étaient bien corrélés avec ESPEN (*k* = 0,555, 0,493), et les valeurs de l’ASC du NRS 2002 et du MNA-SF étaient respectivement de 0,776 et 0,792. *Conclusion* : Cette étude est la première à analyser les patients hospitalisés atteints d’échinococcose à partir de ces outils de dépistage nutritionnel. Nos résultats suggèrent que les méthodes NRS 2002 et MNA-SF sont des outils appropriés pour le dépistage nutritionnel des patients hospitalisés atteints d’échinococcose.

## Introduction

Echinococcosis is a zoonotic parasitic disease. Because of its insidious and asymptomatic early stages, the diagnosis and treatment of echinococcosis is complex, and the disease has a high mortality rate in its late stages. Echinococcosis poses a serious threat to human health as well as social and economic development in susceptible areas [8]. Echinococcosis is prevalent across the world except in Antarctica [25]. There are two kinds of echinococcosis: cystic echinococcosis (CE), which is caused by *Echinococcus granulosus sensu lato*, and alveolar echinococcosis (AE), caused by *Echinococcus multilocularis* [9, 22]. *Echinococcus* is harmful to the human body in many ways, mainly by mechanical damage. Because of the continuous growth of *Echinococcus*, it compresses the surrounding tissues and organs, causing tissue cell atrophy and necrosis, affecting organ function. Patients often have low fever, fatigue, emaciation, loss of appetite and other manifestations [4]. We often find echinococcosis patients with malnutrition in the clinical diagnosis and treatment process. Echinococcosis patients often require prolonged hospitalization and increased costs due to malnutrition. Studies on malnutrition associated with other liver diseases have shown that patients with malnutrition experience higher rates of infection, morbidity and mortality compared to patients without malnutrition [16]. Therefore, studying malnutrition related to hepatic echinococcosis is particularly important. No previous studies have analyzed and evaluated the nutritional status of patients with echinococcosis (as of the start date of this study). In this study, NRS2002 [11], MUST [15], MNA-SF [14] and NRI [5, 7] were used to investigate the nutritional status of hospitalized patients with echinococcosis. Through a comprehensive comparative analysis of the four methods, a suitable nutritional evaluation program was selected for patients with echinococcosis to provide a reference for clinical practice.

## Methods

### Patients

Patients at the Affiliated Hospital of Qinghai University from May 2016 to May 2018 were enrolled as study subjects. All cases were diagnosed as echinococcosis based on the criteria presented in “Expert consensus for the diagnosis and treatment of cystic and alveolar echinococcosis in humans” (2010 edition) [3]. Inclusion criteria for patients were: (i) age over 14 years, (ii) patient is conscious and able to stand, and (iii) patient is willing to participate in the study, and able to answer questions and complete relevant measurements. Exclusion criteria for patients were: (i) hepatic encephalopathy, (ii) difficulty of access to severely ill patients, and (iii) refusal or lack of cooperation with the questionnaire.

### Data collection

General data and anthropometric data of patients were collected from medical records. General data parameters were diagnosis, gender, age, morbidity, appetite change, physical exercise, past medical history, and current combined diseases. Anthropometric parameters included current weight, past weight, height, unexpected weight loss, and body mass index (BMI). The laboratory parameters evaluated were serum albumin.

### Nutritional risk assessment

The following nutritional risk screening tools were used to assess nutritional risk status in patients with echinococcosis: NRS 2002, MNA-SF, MUST and NRI.

NRS-2002 [11] parameters include a disease severity score, nutrition score and age score. The disease severity score is ranked from least to most severe (1–3 points).

Severity of disease score: cirrhosis, hip fracture, long-term hemodialysis, diabetes or chronic disease with acute complications = 1; stroke, major abdominal surgery, hematologic malignancies, or severe pneumonia = 2; head injury, bone marrow transplantation or patients in the intensive care unit with APACHE > 10 (Acute Physiology and Chronic Health Evaluation) = 3. Nutritional score: weight loss of more than 5% in 3 months or food intake is 50–75% of normal expected intake = 1; weight loss of more than 5% in 2 months, BMI of 18.5–20.5 kg/m^2^, or food intake is 25–60% of the normal expected intake = 2; weight loss is more than 5% in 1 month, BMI is <18.5 kg/m^2^, or food intake is < 25% of the expected intake = 3. Age score: age ≥ 70 years = 1; age < 70 years = 0. Nutritional risk was assessed by combining disease severity scores, nutritional scores and age scores. A total score < 3 indicates there is no or low risk of malnutrition, and a total score ≥ 3 indicates a high risk for malnutrition [7, 26].

MNA-SF [14] is an assessment tool designed for elderly subjects based on MNA. It has six parameters related to body mass index, recent weight loss, appetite change, activity ability, psychological stress and neuropsychological problems. Questions cover topics including BMI, recent weight loss, recent acute disease or stress, activity ability, neuropsychiatric disease, recent loss of appetite, dyspepsia, and eating difficulties. The score of each question was 0–2 or 0–3, and 14 was the total score possible. Patients with a score >12 were within a normal nutritional status. Patients with a score ≤11 were at risk of malnutrition [7, 26].

The MUST [1, 15] assessment tool has three clinical parameters: weight, unexpected weight loss, and the presence of acute disease. BMI values > 20, 18.5–20.0 and < 18.5 were assigned scores of 0, 1 and 2, respectively. Presence of acute disease and no acute disease were assigned scores of 0 and 2, respectively. The total risk of malnutrition was determined as follows: 0 score, low risk; 1 score, medium risk; and 2 score, high risk.

NRI [5, 7] is a nutritional risk assessment criterion based on serum albumin concentration and weight loss percentage, as follows: NRI = (1.519 × serum albumin) + (41.7 × current weight/normal weight). NRI score > 100 indicates no risk, 97.5–100 is low risk, 83.5–97.5 is medium risk, and ≤83.5 is high risk.

### New ESPEN malnutrition diagnosis standard

The European Society for clinical nutrition and metabolism (ESPEN) recently proposed a new standard for the diagnosis of malnutrition, which provides a reference standard for the evaluation and comparison of nutrition screening tools. The new ESPEN diagnostic standard includes two options. One is BMI ≤ 18.5 kg/m^2^. The other is weight loss > 5% (in 3 months) or 10% (indefinite amount of time) and reduced BMI (BMI < 20 kg/m^2^ in patients under 70 years old, BMI < 22 kg/m^2^ in patients over 70 years old) [7, 13, 26]. Malnutrition can be diagnosed when the patient meets one of the two options.

#### Statistical analysis

Statistical analysis was performed using SPSS 24.0 (IBM, USA). Continuous variables are expressed as mean and standard deviation (SD), and values for each categorized variable were expressed by frequencies. An independent sample *t*-test, Pearson’s *χ* ^2^ test and Mann–Whitney *U* nonparametric test were used to analyze the differences in variance. In order to analyze the consistency among the four assessment tools, and the consistency between each of the four assessment tools and the new ESPEN malnutrition diagnosis standard [6], kappa (*κ*) statistics were used. The positive and negative likelihood ratios of all four tools were calculated to evaluate their sensitivity and specificity based on the ESPEN criteria for malnutrition diagnosis. Receiver operating characteristic (ROC) curves for the four screening tools were also used to assess the ability to accurately distinguish malnutrition patients.

## Results

The study included 396 patients (164 with AE and 232 with CE). Specific characteristics of the study patients are presented in Table 1. In the CE cohort, 67 patients were malnourished. There were significant differences between the CE patients with and without malnutrition for parameters of age, weight, BMI, ALB and lesion size (*p* < 0.05). No significant differences were observed between the CE patients with and without malnutrition for gender, height, HGB, LYMPH, stage, and number of comorbidities (*p* > 0.05). In the AE cohort, 52 patients were malnourished. There were significant differences between AE patients with and without malnutrition for weight, BMI, ALB, HGB, lesion size and stage (*p* < 0.05). There were no significant differences between the AE patients with and without malnutrition for age, gender, height, LYMPH, and number of comorbidities (*p* > 0.05). There were significant differences between the CE and AE cohorts (*p* < 0.05) related to prevalence of hepatitis B, gallbladder diseases, echinococcosis disseminated.

**Table 1:** Characteristics of patients.

Variable	CE (*n* = 232) ESPEN criteria			AE (*n* = 164) ESPEN criteria		
	Not malnourished (*n* = 165)	Malnourished (*n* = 67)	*p*-value	Not malnourished (*n* = 112)	Malnourished (*n* = 52)	*p-*value
Clinical parameters						
Age	46.91 ± 13.65	37.73 ± 16.78	0.001*	41.91 ± 14.45	37.35 ± 14.87	0.064
Gender						
Male	69	30	0.680	47	23	0.785
Female	96	37		65	29	
Height	1.67 ± 0.15	1.65 ± 0.13	0.341	1.63 ± 0.11	1.62 ± 0.14	0.621
Weight	66.64 ± 10.91	49.09 ± 11.34	<0.001*	61.72 ± 11.27	48.59 ± 8.92	<0.001*
BMI (kg/m^2^)	22.26 ± 2.74	17.74 ± 1.52	<0.001*	23.13 ± 3.09	18.14 ± 1.47	<0.001*
ALB (U/L)	36.22 ± 5.01	32.41 ± 4.67	0.001*	36.67 ± 4.51	32.70 ± 5.10	<0.001*
HGB (g/L)	143.56 ± 24.38	136.74 ± 25.96	0.058	136.06 ± 24.52	120.02 ± 25.29	<0.001*
LYMPH (10^9^/L)	1.71 ± 0.60	1.70 ± 0.75	0.901	1.66 ± 0.68	1.73 ± 0.73	0.574
Lesion size	7.62 ± 3.24	11.81 ± 5.85	<0.001*	10.01 ± 4.51	13.74 ± 4.15	<0.001*
Stage of CE [18]/AE [10]			0.154			0.001*
CE1/I	43	21		29	4	
CE2/II	60	27		25	7	
CE3/IIIa	14	6		9	11	
CE4/IIIb	8	4		18	3	
CE5/IV	40	9		30	26	
Hepatitis B	96	28		49	27	0.047*
Gallbladder diseases	51	30		34	35	0.092
Echinococcosis disseminated [21]	14	9		11	17	0.125
Number of comorbidities			0.106			0.255
0	39	8		22	6	
1–2	77	36		56	25	
3–5	38	16		24	18	
> 5	11	7		10	2	

Abbreviations: BMI, Body Mass Index; ALB, albumin; HGB, hemoglobin; LYMPH, Lymphocyte.

* Values expressing statistical significance (*p* ≤ 0.05).

Table 2 presents the characteristics and anthropometric data of patients with cystic echinococcosis summarized and stratified by nutritional status. There were no statistical differences (*p* > 0.05) in age, height and ALB between the malnutrition and non-malnutrition groups when NRS2002 was used. However, there were significant differences (*p* < 0.05) in gender, weight and BMI between the two groups. There was no statistical difference (*p* > 0.05) in age, gender and height between the two groups when MUST, MNA-SF and NRI were used, but there were statistical differences in weight, BMI and ALB between the two groups. Using the ESPEN criteria, there were no statistical differences (*p* < 0.05) in age, gender, height and ALB between the two groups, and there were statistical differences in weight and BMI between the two groups.

**Table 2:** Characteristics and anthropometric data of cystic echinococcosis by nutritional status.

Patient characteristics	NRS2002			MUST			MNA-SF			NRI		
	No/low risk	High risk	*P*	low risk	Moderate/high risk	*P*	No risk	Risk	*p*	No risk	Risk	*p*
Age	42.47 ± 15.53	42.90 ± 20.51	0.863	41.75 ± 14.24	43.49 ± 20.41	0.449	41.26 ± 15.22	44.20 ± 20.02	0.213	40.20 ± 16.06	44.99 ± 18.86	0.038*
Gender												
Male	46	59	<0.001*	43	56	0.184	48	51	0.258	50	49	0.679
Female	93	41		70	64		75	59		64	70	
Height (cm)	1.63 ± 0.10	1.65 ± 0.14	0.161	1.65 ± 0.08	1.62 ± 0.14	0.064	1.64 ± 0.09	1.63 ± 0.14	0.222	1.64 ± 0.11	1.62 ± 0.12	0.323
Weight (kg)	62.22 ± 12.82	52.50 ± 11.31	<0.001*	65.01 ± 10.90	51.98 ± 11.83	<0.001*	63.53 ± 11.68	52.45 ± 12.16	<0.001*	61.63 ± 13.38	55.11 ± 12.07	<0.001*
BMI (kg/m^2^)	23.30 ± 3.65	19.04 ± 2.36	<0.001*	23.75 ± 3.17	19.54 ± 3.19	<0.001*	23.34 ± 3.37	19.62 ± 3.30	<0.001*	22.62 ± 3.93	20.59 ± 3.43	<0.001*
ALB (U/L)	38.06 ± 5.09	35.93 ± 4.99	0.002*	38.43 ± 4.65	36.04 ± 5.34	<0.001*	38.37 ± 4.87	35.91 ± 5.17	<0.001*	41.29 ± 2.63	33.29 ± 3.74	<0.001*

Abbreviations: BMI, Body Mass Index; ALB, Albumin; NRS 2002, Nutritional Risk Screening 2002; MUST, Malnutrition Universal Screening Tool; MNA-SF, Short Form of Mini Nutritional Assessment; NRI, nutrition risk index; ESPEN, European Society for Clinical Nutrition and Metabolism.

* Values expressing statistical significance (*p* ≤ 0.05).

Table 3 presents the characteristics and anthropometric data of patients with alveolar echinococcosis summarized and stratified by nutritional status. There was no statistical difference in age, gender and height between the two groups when NRS2002 and ESPEN criteria were used, and there were statistical differences in BMI and HGB between the two groups. There were no statistical differences in age, gender and height between the two groups when MUST and MNA-SF were used, and there were statistical differences in weight, BMI and ALB between the two groups. There were no statistical differences in age, gender, height and weight between the two groups in NRI results, and there were statistical differences in ALB and BMI between the two groups. Table 4 lists the consistency analysis results of the three tools with the malnutrition standard. Consistency of *κ* ≥ 0.75 is good; consistency of 0.4 ≤ *κ* ≤ 0.75 is moderate; consistency of *κ* ≤ 0.4 is poor.

**Table 3:** Characteristics and anthropometric data of alveolar echinococcosis by nutritional status.

Patient characteristics	NRS2002			MUST			MNA-SF			NRI		
	No/low risk	High risk	*P*	Low risk	Moderate/high risk	*P*	No risk	Risk	*p*	No risk	Risk	*p*
Age	39.38 ± 13.89	42.98 ± 16.24	0.150	40.43 ± 13.89	40.54 ± 15.50	0.960	39.64 ± 14.12	41.56 ± 15.42	0.410	39.38 ± 15.42	41.02 ± 14.37	0.506
Gender												
Male	45	24	0.330	33	36	0.078	28	41	0.063	21	48	0.627
Female	68	26		58	36		52	42		32	62	
Height (cm)	1.62 ± 0.12	1.64 ± 0.13	0.299	1.62 ± 0.12	1.63 ± 0.12	0.673	1.62 ± 0.11	1.64 ± 0.12	0.240	1.69 ± 0.11	1.70 ± 0.13	0.134
Weight(kg)	59.93 ± 11.83	52.38 ± 11.49	<0.001*	63.41 ± 10.87	52.02 ± 10.75	<0.001*	61.90 ± 11.43	52.19 ± 10.98	<0.001*	60.34 ± 14.09	56.30 ± 11.00	0.070
BMI (kg/m^2^)	22.64 ± 3.31	19.16 ± 2.85	<0.001*	23.88 ± 2.64	19.33 ± 2.81	<0.001*	23.48 ± 2.96	19.16 ± 2.65	<0.001*	23.05 ± 3.72	20.86 ± 3.25	<0.001*
ALB (U/L)	37.44 ± 4.04	30.86 ± 3.97	<0.001*	36.32 ± 4.60	34.56 ± 5.31	0.026*	36.33 ± 4.48	34.28 ± 5.48	0.10	41.04 ± 2.42	32.72 ± 3.49	<0.001*

Abbreviations: BMI, Body Mass Index; ALB, Albumin; NRS 2002, Nutritional Risk Screening 2002; MUST, Malnutrition Universal Screening Tool; MNA-SF, Short Form of Mini Nutritional Assessment; NRI, nutrition risk index; ESPEN, European Society for Clinical Nutrition and Metabolism.

* Values expressing statistical significance (*p* ≤ 0.05).

**Table 4:** Consistency test of three nutritional screening and ESPEN standard.

Nutritional screening tools	Nutritional screening results		AE			CE		
	High risk	No/ low risk	NRS2002	MUST	NRI	NRS2002	MUST	NRI
NRS2002	AE (50)/CE (94)	AE (113)/CE (139)			*K* = 0.330			*K* = 0.222
MUST	AE (83)/CE (120)	AE (80)/CE (113)	*K* = 0.403		*K* = 0.115	*K* = 0.516		*K* = 0.253
NRI	AE (72)/CE (119)	AE (91)/CE (114)	*K* = 0.330	*K* = 0.115		*K* = 0.222	*K* = 0.253	
MNA-SF	AE (110)/CE (109)	AE (53)/CE (124)	*K* = 0.409	*K* = 0.645	*K* = 0.128	*K* = 0.462	*K* = 0.709	*K* = 0.245

Abbreviations: NRS 2002, Nutritional Risk Screening 2002; MUST, Malnutrition Universal Screening Tool; MNA-SF, Short Form of Mini Nutritional Assessment; NRI, nutrition risk index; ESPEN, European Society for Clinical Nutrition and Metabolism.

According to the new ESPEN diagnostic standard, the sensitivity and specificity of the four assessed nutritional screening tools are inconsistent. In cystic echinococcosis patients, MUST was the most sensitive (91.1%) tool and NRI was the least sensitive (66.1%) compared with ESPEN. NRS2002 had the highest specificity (75.8%), while NRI had the lowest specificity (55.1%). MUST had the highest negative predictive value (94.3%), while NRI had the lowest negative predictive value (79.8%). Finally, the area-under-the-curve (AUC) calculated by ROC showed that NRS 2002, MUST and MNA-SF had a moderate diagnostic value (AUC values for MUST, NRS 2002 and MNA-SF were 0.776, 0.780 and 0.803, respectively), while NRI had poor diagnostic value (AUC was 0.607). The results are detailed in Table 5.

**Table 5:** Comparison of four screening tools for malnutrition with ESPEN diagnostic criteria in cystic echinococcosis patients.

Nutritional screening tools	Nutritional screening results	ESPEN criteria		Sensitivity (%)	Specificity (%)	Positive predictive value (%)	Negative predictive value (%)	Positive likelihood ratio (LR +)	Negative predictive value (LR −)	*K* value	AUC
		Malnourished	Not malnourished								
NRS2002	High risk (94)	54	40	79.4	75.8	57.4	89.9	3.28	0.27	0.496	0.776
	No/low risk (139)	14	125								
MUST	High risk (120)	62	58	91.1	64.8	51.7	94.6	2.59	0.14	0.457	0.780
	No/low risk (113)	6	107								
MNA-SF	High risk (109)	61	48	89.7	70.9	55.9	94.3	3.08	0.14	0.515	0.803
	No/low risk (124)	7	117								
NRI	High risk (119)	45	74	66.1	55,1	37.8	79.8	1.48	0.61	0.175	0.607
	No/low risk (114)	23	91								

Abbreviations: NRS 2002, Nutritional Risk Screening 2002; MUST, Malnutrition Universal Screening Tool; MNA-SF, Short Form of Mini Nutritional Assessment; NRI, nutrition risk index; ESPEN, European Society for Clinical Nutrition and Metabolism; AUC, area-under-the-curve from ROC.

In alveolar echinococcosis patients, MNA-SF had the highest sensitivity (86.2%) compared with ESPEN, while NRS2002 had the lowest sensitivity (68.6%). NRS2002 had the highest specificity (86.6%), while NRI had the lowest sensitivity (40.2%). MUST and MNA-SF had the highest negative predictive value (91.2%), while NRI had the lowest negative predictive value (84.9%). Finally, the area-under-the-curve (AUC) calculated using ROC showed that NRS 2002, MUST and MNA-SF had moderate diagnostic value (AUC values of NRS 2002, MUST and MNA-SF are 0.776, 0.757 and 0.792, respectively), while NRI had poor diagnostic value (AUC is 0.622). The results are detailed in Table 6.

**Table 6:** Comparison of four screening tools for malnutrition with ESPEN diagnostic criteria in alveolar echinococcosis patients.

Nutritional screening tools	Nutritional screening results	ESPEN criteria		Sensitivity (%)	Specificity (%)	Positive predictive value (%)	Negative predictive value (%)	Positive likelihood ratio (LR+)	Negative predictive value (LR−)	*K* value	AUC
		Malnourished	Not malnourished								
NRS2002	High risk (50)	35	15	68.6	86.6	70.0	85.8	5.12	0.36	0.555	0.776
	No/low risk (113)	16	97								
MUST	High risk (83)	43	29	84.3	74.1	59.7	91.2	3.26	0.21	0.525	0.757
	No/low risk (80)	8	83								
MNA-SF	High risk (72)	44	39	86.2	65.1	53.0	91.2	2.48	0.21	0.439	0.792
	No/low risk (91)	7	73								
NRI	High risk (110)	43	67	84.3	40.2	39.1	84.9	1.41	0.39	0.186	0.622
	No/low risk (53)	8	45								

Abbreviations: NRS 2002, Nutritional Risk Screening 2002; MUST, Malnutrition Universal Screening Tool; MNA-SF, Short Form of Mini Nutritional Assessment; NRI, nutrition risk index; ESPEN, European Society for Clinical Nutrition and Metabolism; AUC, area under the curve from ROC.

## Discussion

Echinococcosis, a type of chronic consumptive disease, can damage the liver continuously and oppress normal liver tissue, and surrounding tissues and organs. It can lead to malnutrition and emaciation [22]. Echinococcosis is usually found in the liver, but can also be transferred to the abdominal cavity, lungs, brain and other organs [19, 20, 24]. It has the characteristics of slow onset and occult onset. At present, there are few reports on the nutritional status of patients with echinococcosis. In this study, the nutritional status of patients with alveolar echinococcosis or cystic echinococcosis (hydatid cysts and hydatid vesicles) was analyzed comprehensively for the first time. Four common nutritional screening tools were used to evaluate echinococcosis, and the results were compared with the results of the new European Society for clinical nutrition and metabolism (ESPEN) diagnostic standard [13, 26] to assess their suitability for diagnosing malnutrition in patients with echinococcosis disease. According to the ESPEN diagnostic criteria, 29.2% of the patients with cystic echinococcosis and 31.1% of the patients with alveolar echinococcosis were malnourished.

Malnutrition in patients with CE may be caused by the cystic hydatid cyst, which continuously increases in volume, putting pressure on the liver parenchyma and the bile duct. Bile duct necrosis occurs under a long-term high-pressure external force, resulting in the occurrence of cysts, obstructive jaundice, cholangitis, secondary infection of cyst, abnormal liver function, and the imbalance of nutrient metabolism [3]. Through asexual proliferation and strong granuloma reaction, AE infiltrates and grows to surrounding tissues, which is similar to a tumor to a certain extent, thus causing serious pathological damage to normal cells and tissues of the liver, compressing and eroding the bile duct, leading to extensive fibrosis, infiltration and necrosis of various inflammatory cells [2, 23]. Our study found that in-patients with echinococcosis often have other diseases as well. In this study, 46.2% of patients with echinococcosis also had hepatitis B, and 37.9% had gallbladder diseases. Echinococcosis is most prevalent in the Tibet Autonomous Region of China. There is also a high incidence rate of hepatitis B (HBV) among these populations, which may be related to poor living environments in some cases. Some studies have shown that the incidence rate of HBV in Tibetan populations is related to poor hygiene conditions, such as diet and drinking water, and lack of awareness of disease prevention methods and local epidemics [12]. Hepatitis B can lead to anorexia and daily calorie intake declines in patients with chronic liver disease, resulting in malnutrition [17]. In the same way, patients with cholecystitis may suffer from malnutrition due to the reduction of food intake and dyspepsia [16]. These may be additional reasons for the high incidence of malnutrition in hospitalized echinococcosis patients. In this study, malnutrition in both the AE and CE patients was associated with larger lesion sizes (statistically significant difference). This indicates that lesion size may be a risk factor for malnutrition in patients with echinococcosis. For patients with AE, the classification level may also be a risk factor. Nonparametric analysis results showed that patients with higher echinococcosis classification were more likely to suffer from malnutrition.

In this study, according to NRS2002 and MUST results, 40.3% and 51.5% of patients with CE were found to be at moderate or high risk of malnutrition. Using MNA-SF and NRI, results showed that 46.8% and 51.1% of patients, respectively, were found to be at risk of malnutrition. There were statistically significant differences in how the four nutritional screening tools classified patients with cystic echinococcosis by nutritional risk. This may be attributed to the differences in the nutritional screening tools. Among these tools, the reason for the poor consistency between NRI and the other three tools may be that many in-patients with cystic echinococcosis also have other diseases such as hepatitis, infections, etc., which lead to decreases in albumin and affect the NRI score. In a study by Poulia et al. [13], a comparison of NRS2002 and MUST tools was performed for hospitalized patients, using ESPEN diagnostic criteria as the gold standard of malnutrition. In this study, the new diagnostic criteria for malnutrition of MUST and ESPEN were better correlated (*k* = 0.843). However, in our study of patients with hydatid cysts, the correlation analysis comparing the four screening tools to the ESPEN diagnostic criteria showed that the correlation for MUST, NRS2002 and MNA-SF was moderate (*k* = 0.457, 0.496 and 0.515, respectively), and the correlation between ESPEN and NRI was poor (*k* = 0.175).

In this study, according to NRS2002 and MUST, 30.7% and 50.9% of the patients, respectively, with AE were found to be at moderate or high risk of malnutrition. Using MNA-SF and NRI, 44.1% and 67.4% of patients, respectively, were found to be at risk of malnutrition. There were statistically significant differences in how the four nutritional screening tools classified patients with alveolar echinococcosis by nutritional risk. Ye et al. [26] reported a comparison of NRS2002, MUST and MNA-SF tools in elderly patients with gastrointestinal cancer, using ESPEN diagnostic criteria as the gold standard of malnutrition. Their results showed that compared with NRS 2002 and MNA-SF, the correlation between MUST and ESPEN diagnostic criteria was the best (К = 0.530). In the current study of patients with AE, the correlation analysis between the four screening tools and ESPEN diagnostic criteria showed that the correlations between ESPEN and MUST, and NRS2002 and MNA-SF, respectively, were moderate (*k* = 0.525, 0.555, 0.439), and the correlation between ESPEN and NRI was poor (*k* = 0.186).

According to ESPEN diagnostic criteria and the four nutrition screening tools, AE and CE patients vary in incidence of malnutrition, with AE patients exhibiting a slightly higher rate of malnutrition than CE patients. Some patients with both of these types of echinococcosis had disseminated echinococcosis. In this study, 17.1% of AE patients and 9.9% of CE patients had disseminated, which may be one of the reasons the AE patients had a slightly higher incidence of malnutrition. In CE patients, the consistency between MNA-SF and ESPEN results was the best, while in AE, the consistency between NRS2002 and ESPEN results was the best. The purpose of nutrition screening is to accurately identify patients who are malnourished or at risk of malnutrition, and who can benefit from nutrition therapy. Good nutritional screening should be highly sensitive and specific. In this study of CE, according to the ESPEN diagnostic criteria, although the AUC value (0.780) of MUST was slightly higher than that of NRS2002 (0.776), the positive likelihood ratio of NRS2002 was significantly higher than that of MUST. In this study of AE, although the AUC value of MNA-SF was higher (0.792) than that of NRS2002 (0.776), the positive likelihood ratio and recessive likelihood ratio of NRS2002 were significantly higher than the corresponding values for MNA-SF. Based on these results, we conclude that MNA-SF and NRS2002 can be used in patients with CE and AE, but further research is needed to confirm this.

This study had some limitations. First, the scope of this study was hospitalized patients with hydatidosis, with many complications, which may not accurately represent all patients with echinococcosis, and the risk factors of malnutrition in patients with echinococcosis may not be comprehensive. Second, the sample size was relatively small, and focused on a single center. Third, this study lacks the reduction of fat free mass index (FFMI) to diagnose malnutrition. The ESPEN malnutrition diagnosis standard can also allow diagnosis by unintended weight loss and fat free mass index (FFMI) reduction. The hospital where our study was focused lacked the specialized equipment needed for FFMI measurement. Therefore, further research is needed to verify our findings.

## Conclusions

This is the first time common nutritional screening tools have been used to screen the nutritional risk of echinococcosis patients and the first comparison of four malnutrition screening tools (NRS 2002, MUST, MNA-SF and NRI) against the ESPEN malnutrition diagnosis standard. In this study, according to the ESPEN diagnostic criteria for malnutrition in patients with CE and AE, the malnutrition rates were 29.2% and 31.1%, respectively. NRS2002 and MNA-SF may be better screening tools for hospitalized patients with hepatic echinococcosis.

## Conflicts of interest

The authors have no potential conflict of interest.

## Funding

Funding were provided by Innovation platform construction project in the Qinghai Department of Science and Technology (2020-ZJ-Y01) and the Key Projects of Precision Medicine Research in National Key R&D Programmes (2017YFC0909900).
